# Influence of the Triglyceride-Glucose Index on Adverse Cardiovascular and Cerebrovascular Events in Prediabetic Patients With Acute Coronary Syndrome

**DOI:** 10.3389/fendo.2022.843072

**Published:** 2022-02-22

**Authors:** Qianyun Guo, Xunxun Feng, Bin Zhang, Guangyao Zhai, Jiaqi Yang, Yang Liu, Yuyang Liu, Dongmei Shi, Yujie Zhou

**Affiliations:** ^1^ Beijing Key Laboratory of Precision Medicine of Coronary Atherosclerotic Disease, Clinical Center for Coronary Heart Disease, Department of Cardiology, Beijing Anzhen Hospital, Beijing Institute of Heart Lung and Blood Vessel Disease, Capital Medical University, Beijing, China; ^2^ Department of Cardiology, Fuwai Hospital, National Center for Cardiovascular Disease, Chinese Academy of Medical Science and Peking Union Medical College, Beijing, China

**Keywords:** TyG index, prediabetes, ACS, prognosis, MACCEs

## Abstract

**Background:**

Cardiovascular disease and insulin resistance are closely related. The triglyceride-glucose (TyG) index is frequently used as an indicator of insulin resistance. However, there is scant information on the TyG index in the prediabetic population, nor is the prognostic significance of the index known for prediabetes and acute coronary syndrome (ACS) patients.

**Methods:**

The clinical endpoint was a major adverse cardiovascular and cerebrovascular event (MACCEs), including cardiac-related death, non-fatal myocardial infarction, ischemia-driven revascularization, and stroke. The TyG index was calculated as = ln [(triglyceride level, mg/dL) × (glucose level, mg/dL)÷2] under fasting conditions.

**Results:**

The study included 2,030 prediabetic patients with ACS. Patients were followed up for 2.5 years, during which the total incidence of MACCEs was 12%. After adjustment for covariates, the TyG index was found to be predictive of prediabetes with ACS (HR 4.942, 95%CI: 3.432-6.115, P<0.001). Using propensity score matching, 574 pairs were successfully matched, and the two groups were analyzed in terms of survival. This showed that there was a significantly greater incidence of MACCEs in patients with high TyG indices (HR 3.526, 95%CI: 2.618-4.749, P<0.001), mainly due to ischemia-driven revascularization and stroke.

**Conclusions:**

The TyG index independently predicts future MACCEs and may be an important prognostic indicator for patients with prediabetes and ACS.

## Background

The prevalence of diabetes has risen from 108 million to 422 million in the last thirty years, of which type 2 diabetes (T2DM) accounts for more than 90%, and studies estimate that it will increase to 642 million by 2040. Most patients go through a prediabetic stage before they develop diabetes (1, 2). The prevalence of prediabetes is also rising globally. Research shows that by 2030, more than 470 million people will suffer from prediabetes. Prediabetes is associated with co-existing insulin resistance (IR) and β-cell dysfunction, and these abnormalities begin before blood sugar changes are detected. Prediabetes is a complex, multi-factorial metabolic disorder, and its pathophysiology centers around IR, impaired incretin action, and high insulin secretion. Observational evidence has linked prediabetes with an elevated risk of nephropathy, diabetic retinopathy, and cardiovascular diseases (CVDs) (3–5).

IR is usually present in prediabetes, before development to T2DM (6). It has been found that the triglyceride-glucose (TyG) index is a practical method of evaluating IR, and the TyG index has been verified in multiple populations around the world, and its effectiveness and reliability was consistently showed in IR detecting (7, 8). Recent studies suggest that prediabetes is correlated with an increased risk of T2DM, CVD, dementia, and cancer; moreover, its incidence is increasing worldwide (9). In China, the prevalence of prediabetes rose from 15.5% in 2008 to 35.2% in 2017, with about 357 million people suffering from prediabetes (10, 11). Notably, the best control of glycemia and IR as in the case of metformin therapy could result in these patients in best clinical outcomes (12, 13). Moreover, asymptomatic diabetic patients with high TyG indices have a higher risk of coronary artery stenosis (14). Therefore, the TyG index may be a valuable biomarker for the development of diabetes (15, 16), allowing the effective screening and early detection of individuals at high risk for T2DM (17). In addition, it has been found that after adjustment for confounders, there was a close relationship between the TyG index and prediabetes (18).

In addition, the TyG index, which also measures IR, may be helpful for the early recognition of cardiovascular events, with higher TyG indices in high-risk groups related to an increased risk of CVD (19). Although there has been an increase in the number of studies on the TyG index and CVDs recently, there is still a lack of prognosis-related studies on acute coronary syndrome (ACS) in prediabetic patients. Identification of an effective means of evaluating the prognosis of prediabetic patients with ACS would assist the recognition of those at high risk of major adverse cardiovascular and cerebrovascular events (MACCEs) for closer monitoring or potential early intervention. Based on the results of follow-up, we aimed to explore the relationship between the prognosis and the TyG index in prediabetic patients with ACS.

## Methods

### Study Design, Patient Population and Definitions

**Figure 1:**
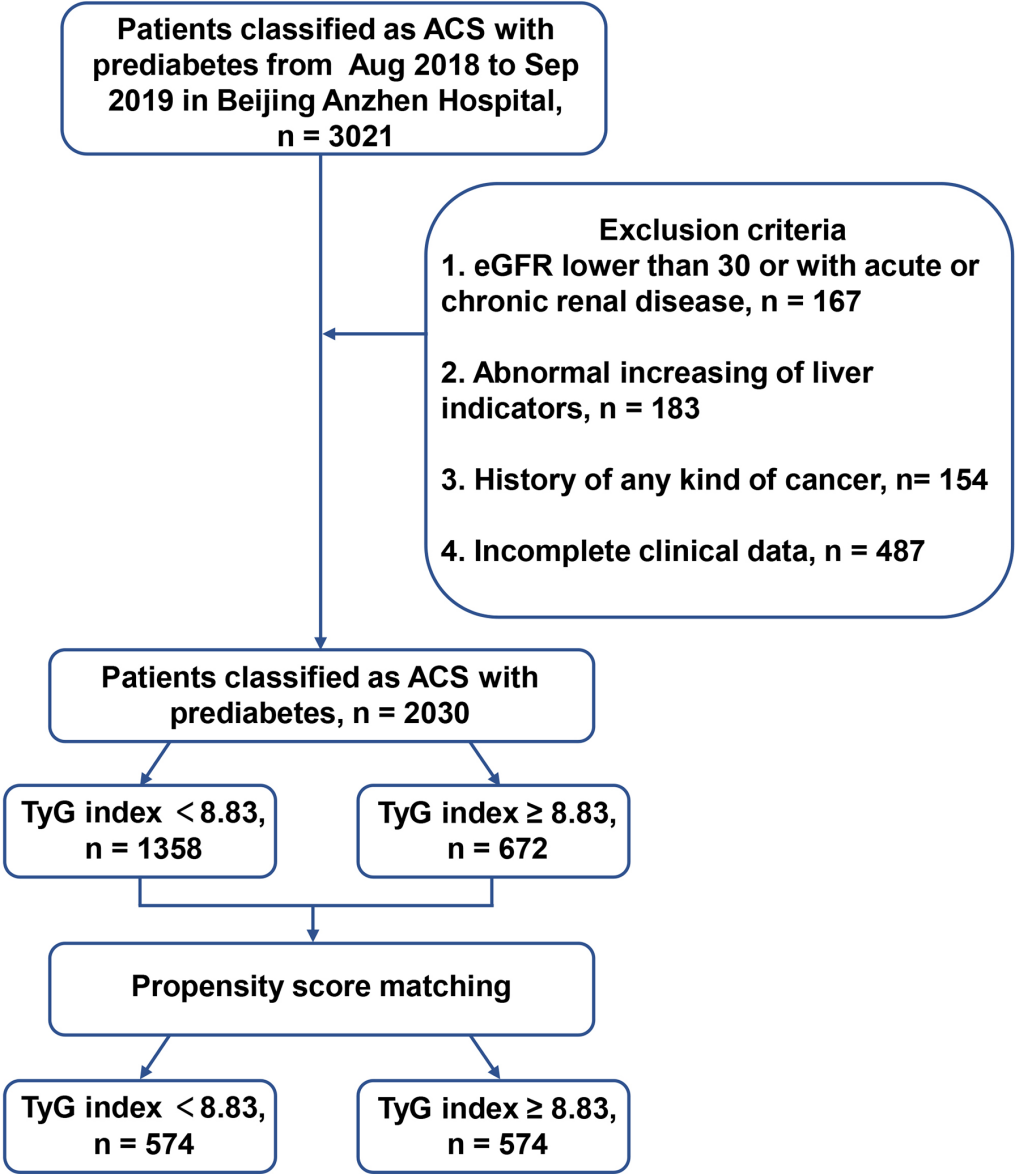
The flow chart of enrolled patients. ACS, acute coronary syndrome; HbA1c, glycosylated hemoglobin A1c; DM, diabetes mellitus; eGFR, estimated glomerular filtration rate; TyG, triglyceride-glucose.

**Figure 2:**
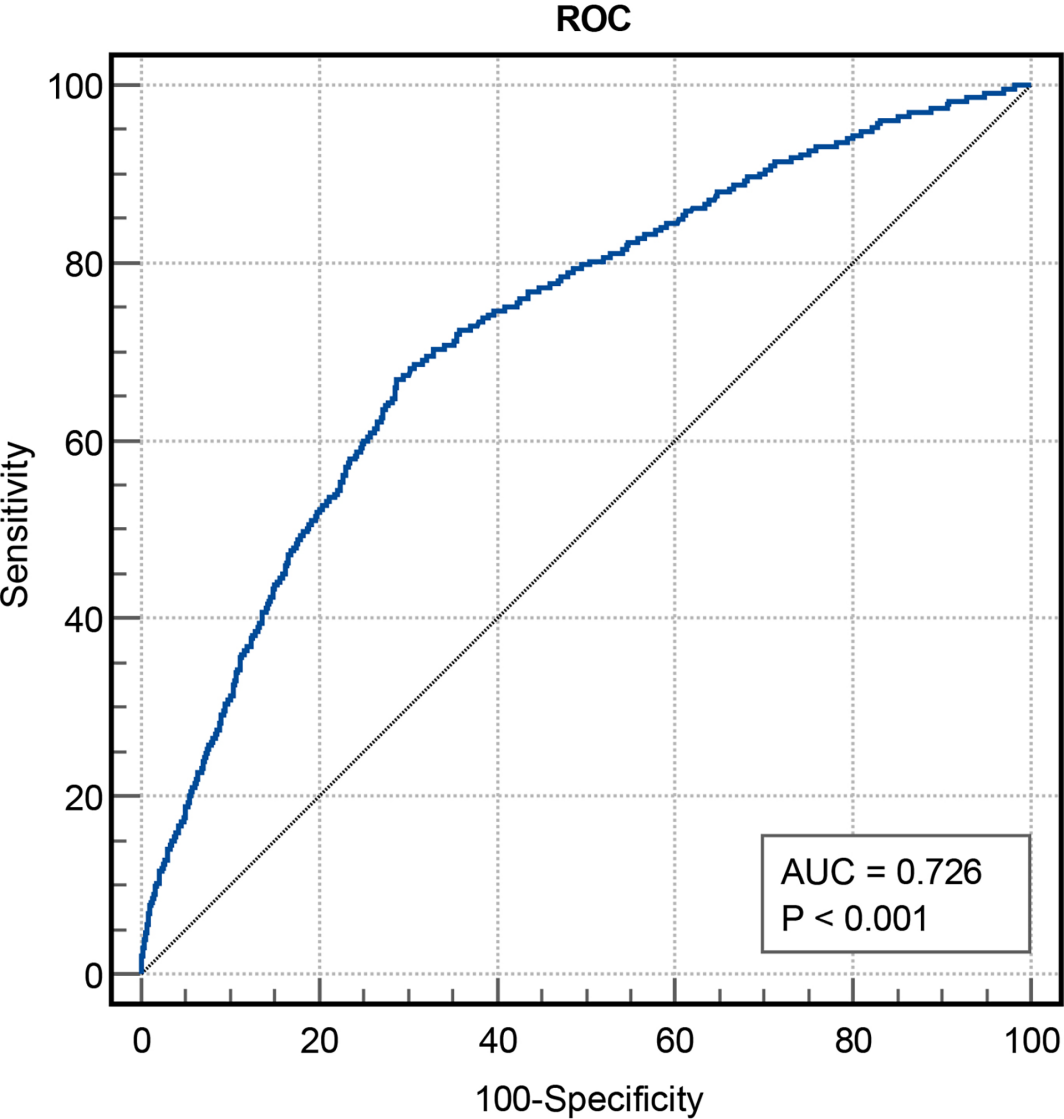
The predictive values of TyG index for the risk of MACCEs. TyG, triglyceride-glucose; MACCEs, major adverse cardiovascular and cerebrovascular events; AUC, area under curve.

This single center, retrospective, observational study enrolled 2030 patients with prediabetes and ACS admitted to Anzhen Hospital for coronary angiography from August 2018 to September 2019. Using the definition of prediabetes, we discussed and reviewed previous studies and found that although the new glycosylated hemoglobin A1c (HbA1c) criteria identified fewer high-risk individuals than those with impaired fasting glucose, HbA1c (in the range of 5.7-6.4%) had a similar the predictive value to impaired fasting glucose alone (20). At the same time, considering that the fasting glucose of patients admitted to hospital may be affected by their diet, the final definition was based on the 2021 AHA Classification and Diagnosis of Diabetes, with an HbA1c range from 5.7% to 6.4% (21). ACS was defined based on appropriate guidelines, including unstable angina pectoris (UAP), ST-segment elevation myocardial infarction (STEMI) and non-ST-segment elevation myocardial infarction (NSTEMI) (22). The exclusion criteria were patients with (1) abnormal liver function: severe insufficiency with alanine transaminase (ALT) or aspartate transaminase (AST) over 5 upper limit of normal; abnormal kidney function: with estimated glomerular filtration rate (eGFR) < 30 mL/(min * 1.73 m^2^); (2) incomplete baseline and follow-up clinical data; (3) a history of coronary artery bypass grafting (CABG); (4) any kind of cancer or other major diseases affecting long-term survival. The TyG index cut-off was calculated using the receiver operating characteristic (ROC) curve (TyG index = 8.83). The patients were assigned to two groups based on their TyG indices, the “high TyG index group” (TyG index ≥ 8.83) and the “low TyG index group” (TyG index < 8.83). Patients were matched through the 1:1 propensity score between the two groups, and 574 pairs were successfully identified for survival analysis. These details are shown in the flow chart (**Figures 1** and **2**).

### Data Collection and Laboratory Examination

We performed the data collection of clinical characteristics using case report form, including age, gender, body mass index (BMI), systolic blood pressure (SBP), diastolic blood pressure (DBP), previous medical history, laboratory examination and types of medication taken. The TyG index was defined as previously reported: TyG index = ln (fasting triglyceride (TG, mg/dL) × fasting blood glucose level (FBG, mg/dL)/2) (23). The collection of peripheral venous blood samples was performed in the morning after an overnight fast and immediately sent for analysis to the central laboratory methods of Beijing Anzhen Hospital. The analysis included TG, total cholesterol (TC), low-density lipoprotein cholesterol (LDL-C), high density lipoprotein cholesterol (HDL-C), creatinine (Cr), serum uric acid (SUA), FBG, HbA1c, C-reactive protein (CRP), high sensitivity troponin I (hs-TNI), amongst other hematological and biochemical parameters. All of the above biochemical variables were evaluated at baseline.

### Definition of Clinical Endpoints

All patients were routinely followed up at 3, 6 and 12 months, and then annually for 30 months. Information on adverse events was gained from patients or their families by telephone questionnaires. New-onset MACCEs was defined as the primary endpoint comprising cardiac-related death, non-fatal myocardial infarction (MI), ischemia-driven revascularization, and stroke. Secondary endpoints included the same events (24, 25), which were recorded for each patient during the 2.5-year follow-up after discharge. The first primary endpoint event that occurred during the follow-up was used for analysis. For multiple adverse outcomes during the follow-up, only the most severe event was used (cardiac death > MI/stroke > ischemia-driven revascularization).

### Statistical Analysis

Normally distributed continuous variables were represented as means ± standard deviation, while those with skewed distribution were shown as median (interquartile range [IQR]). Besides, the continuous variables between two groups were compared using one-way analysis of variance or the Kruskal-Wallis test, and the categorical variables were showed as frequencies and analyzed using test of chi-square. The Pearson and Spearman correlation analyses were applied to assess the relationship between two variables as appropriate. Time dependent Cox proportional hazards regression was performed to evaluate the hazard ratios (HRs) for MACCEs associated with the predictive values of TyG and variables. In multivariate analyses, in addition to age, gender (reference to male), BMI, SBP, DBP, smoking status, history of hypertension and hyperlipemia, levels of LDL-C, HDL-C, Cr, SUA, eGFR, brain natriuretic peptide (BNP), and CRP were adjusted. Restricted cubic spline (RCS) regression was used to show the graphical association between TyG index and MACCEs. ROC curves were used to assess the cut-off point of the TyG index for MACCEs. To match the patients between the high-TyG index and low-TyG index groups, a 1:1 propensity score-matched analysis was conducted (details in **Supplementary Material**). Kaplan–Meier analysis was performed to evaluate the time-related events and the evaluation of discrepancies was assessed by log-rank tests. Two-tailed P-values < 0.05 were regarded as statistically significant. All statistical analyses in this study used MedCalc version 20.0.3, SPSS version 22.0 and R version 4.0.0.

## Results

### Baseline Characteristics

In total, 2030 patients were enrolled in the study. During 2.5 years of follow-up, 233 (11.5%) of the 2030 patients experienced MACCEs, including 11 (0.5%) cardiac deaths, 29 (1.4%) non-fatal MIs, 180 (8.9%) ischemia-driven revascularizations and 41 (2.0%) strokes. Using stratification based on MACCEs occurrence, the baseline clinical characteristics of the overall population were grouped into MACCEs and non-MACCEs (**Table 1**). The TyG index levels in the MACCEs group (9.00 ± 0.51) were significantly elevated compared to those in the non-MACCEs group (8.59 ± 0.48) (P<0.001). The two groups also differed significantly in terms of TC, TG, HDL-C, Cr, SUA, FBG, HbA1c, hs-TNI, and use of angiotensin-converting enzyme inhibitor (ACEI) medication (P<0.05) but not in terms of other indicators. The area under the curve (AUC) of the TyG index for MACCEs was 0.726 (95% CI 0.691–0.761, P < 0.001, P<0.001). The TyG index of 8.83 was determined as the optimal cutoff point for predicting MACCEs with a sensitivity of 64.4% and a specificity of 71.7%. Patients with raised TyG indices tended seemed to be younger, with high BMI and DBP levels and a greater incidence of hypertension compared to the lower TyG index group. Besides these, the levels of LDL-C, TC, TG, Cr, SUA, FBG, and β-blocker use were higher in the group with high TyG indices (P<0.01), while the HDL-C levels were lower in the group with low TyG indices (P<0.001) (**Table 2**).

**Table 1:** Baseline clinical characteristics among the MACCEs and non-MACCEs group of overall population.

Characteristics	Overall (n=2030)	Non-MACCEs (n=1797)	MACCEs (n=233)	P
**Demographic**				
**Age, years**	58.87 ± 10.27	59.02 ± 10.29	57.74 ± 10.06	0.074
**Gender, (male%)**	1505 (74.1)	1327 (73.8)	178 (76.4)	0.449
**BMI, kg/m^2^**	25.64 ± 3.31	25.59 ± 3.34	25.97 ± 3.08	0.107
**SBP, mmHg**	128.42 ± 16.83	128.46 ± 16.84	128.06 ± 16.77	0.735
**DBP, mmHg**	76.91 ± 10.86	76.86 ± 10.76	77.26 ± 11.63	0.603
**Medical history, n (%)**				
**Smoking**	1026 (50.5)	902 (50.2)	124 (53.2)	0.424
**Hypertension**	1214 (59.8)	1074 (59.8)	140 (60.1)	0.982
**Hyperlipemia**	1412 (69.6)	1252 (69.7)	160 (68.7)	0.813
**Pre-PCI**	479 (23.6)	425 (23.7)	54 (23.2)	0.937
**Laboratory results**				
**LDL-C, mmol/L**	2.41 ± 0.84	2.40 ± 0.85	2.46 ± 0.84	0.323
**TC, mmol/L**	4.04 ± 0.98	4.03 ± 0.98	4.16 ± 1.02	0.046
**TG, mmol/L**	1.38 ± 0.71	1.32 ± 0.64	1.86 ± 0.96	<0.001
**HDL-C, mmol/L**	1.14 ± 0.26	1.15 ± 0.27	1.07 ± 0.22	<0.001
**TyG index**	8.64 ± 0.50	8.59 ± 0.48	9.00 ± 0.51	<0.001
**Cr, μmol/L**	70.86 ± 14.80	70.61 ± 14.54	72.82 ± 16.58	0.032
**SUA, μmol/L**	355.37 ± 88.15	352.97 ± 85.75	373.86 ± 103.20	0.001
eGFR, mL/(min* 1.73 m^2^)	96.48 ± 13.09	96.50 ± 12.98	96.34 ± 13.94	0.858
**BNP, pg/mL**	47.33 ± 102.55	47.35 ± 101.94	47.16 ± 107.36	0.979
**CRP, mg/L**	3.17 ± 5.56	3.13 ± 5.55	3.48 ± 5.63	0.369
**FBG, mmol/L**	5.83 ± 1.26	5.77 ± 1.21	6.25 ± 1.56	<0.001
**HbA1c, %**	6.00 ± 0.24	5.99 ± 0.24	6.05 ± 0.24	0.002
**hs-TNI, pg/mL**	0.36 ± 2.42	0.30 ± 2.12	0.80 ± 4.02	0.003
**Clinical presentation, n (%)**				
**STEMI**	118 (5.8)	98 (5.5)	20 (8.6)	0.076
**NSTEMI**	108 (5.3)	94 (5.2)	14 (6.0)	0.732
**UAP**	1804 (88.9)	1605 (89.3)	199 (85.4)	0.094
**Medication, n (%)**				
**Antiplatelet**	2029 (100.0)	1796 (99.9)	233 (100.0)	1.000
**Statin**	2026 (99.8)	1793 (99.8)	233 (100.0)	1.000
**ACEI**	337 (16.6)	284 (15.8)	53 (22.7)	0.010
**ARB**	1504 (74.1)	1320 (73.5)	184 (79.0)	0.084
**β-blocker**	1608 (79.2)	1422 (79.1)	186 (79.8)	0.872
**Nitrate**	1841 (90.7)	1628 (90.6)	213 (91.4)	0.775

BMI, body mass index; SBP, systolic blood pressure; DBP, diastolic blood pressure; PCI, percutaneous coronary intervention; LDL-C, low-density lipoprotein cholesterol; TC, total cholesterol; TG, triglyceride; HDL-C, high density lipoprotein cholesterol; TyG, triglyceride-glucose; Cr, creatinine; SUA, serum uric acid; eGFR, estimated glomerular filtration rate; BNP, brain natriuretic peptide; CRP, C-reactive protein; FBG, fasting blood glucose; HbA1c, glycosylated hemoglobin A1c; hs-TNI, high sensitivity troponin I; STEMI, ST-segment elevation myocardial infarction; NSTEMI, non-ST-segment elevation myocardial infarction; UAP, unstable angina pectoris; ACEI, angiotensin-converting enzyme inhibitor; ARB, angiotensin receptor blocker.

**Table 2:** Baseline clinical characteristics of patients stratified by the optimal cutoff point of TyG index.

Characteristics	Overall (n=2030)	Lower TyG index (<8.83, n=1358)	Higher TyG index (≥8.83, n=672)	P
**Demographic**				
**Age, years**	58.87 ± 10.27	59.70 ± 10.17	57.21 ± 10.28	<0.001
**Gender, (male%)**	1505 (74.1)	1021 (75.2)	484 (72.0)	0.140
**BMI, kg/m^2^**	25.64 ± 3.31	25.39 ± 3.35	26.14 ± 3.18	<0.001
**SBP, mmHg**	128.42 ± 16.83	128.45 ± 17.02	128.35 ± 16.45	0.907
**DBP, mmHg**	76.91 ± 10.86	76.52 ± 10.92	77.69 ± 10.72	0.022
**Medical history, n (%)**				
**Smoking**	1026 (50.5)	689 (50.7)	337 (50.1)	0.840
**Hypertension**	1214 (59.8)	791 (58.2)	423 (62.9)	0.047
**Hyperlipemia**	1412 (69.6)	939 (69.1)	473 (70.4)	0.603
**Pre-PCI**	479 (23.6)	337 (24.8)	142 (21.1)	0.074
**Laboratory results**				
**LDL-C, mmol/L**	2.41 ± 0.84	2.30 ± 0.82	2.63 ± 0.85	<0.001
**TC, mmol/L**	4.04 ± 0.98	3.88 ± 0.93	4.37 ± 1.00	<0.001
**TG, mmol/L**	1.38 ± 0.71	1.05 ± 0.32	2.05 ± 0.79	<0.001
**HDL-C, mmol/L**	1.14 ± 0.26	1.18 ± 0.27	1.06 ± 0.23	<0.001
**TyG index**	8.64 ± 0.50	8.37 ± 0.33	9.17 ± 0.31	<0.001
**Cr, μmol/L**	70.86 ± 14.80	70.24 ± 14.10	72.12 ± 16.05	0.007
**SUA, μmol/L**	355.37 ± 88.15	345.40 ± 82.50	375.50 ± 95.54	<0.001
eGFR, mL/(min* 1.73 m^2^)	96.48 ± 13.09	96.55 ± 12.50	96.35 ± 14.21	0.742
**BNP, pg/mL**	47.33 ± 102.55	49.61 ± 104.01	42.72 ± 99.47	0.155
**CRP, mg/L**	3.17 ± 5.56	3.10 ± 5.66	3.31 ± 5.36	0.419
**FBG, mmol/L**	5.83 ± 1.26	5.54 ± 0.97	6.40 ± 1.56	<0.001
**HbA1c, %**	6.00 ± 0.24	6.00 ± 0.24	6.00 ± 0.24	0.763
**hs-TNI, pg/mL**	0.36 ± 2.42	0.31 ± 2.40	0.45 ± 2.46	0.215
**Clinical presentation, n (%)**				
**STEMI**	118 (5.8)	79 (5.8)	39 (5.8)	0.999
**NSTEMI**	108 (5.3)	68 (5.0)	40 (6.0)	0.431
**UAP**	1804 (88.9)	1211 (89.2)	593 (88.2)	0.580
**Medication, n (%)**				
**Antiplatelet**	2029 (100.0)	1358 (100.0)	671 (99.9)	0.719
**Statin**	2026 (99.8)	1354 (99.7)	672 (100.0)	0.381
**ACEI**	337 (16.6)	212 (15.6)	125 (18.6)	0.101
**ARB**	1504 (74.1)	1020 (75.1)	484 (72.0)	0.150
**β-blocker**	1608 (79.2)	1049 (77.2)	559 (83.2)	0.002
**Nitrate**	1841 (90.7)	1235 (90.9)	606 (90.2)	0.634

BMI, body mass index; SBP, systolic blood pressure; DBP, diastolic blood pressure; PCI, percutaneous coronary intervention; LDL-C, low-density lipoprotein cholesterol; TC, total cholesterol; TG, triglyceride; HDL-C, high density lipoprotein cholesterol; TyG, triglyceride-glucose; Cr, creatinine; SUA, serum uric acid; eGFR, estimated glomerular filtration rate; BNP, brain natriuretic peptide; CRP, C-reactive protein; FBG, fasting blood glucose; HbA1c, glycosylated hemoglobin A1c; hs-TNI, high sensitivity troponin I; STEMI, ST-segment elevation myocardial infarction; NSTEMI, non-ST-segment elevation myocardial infarction; UAP, unstable angina pectoris; ACEI, angiotensin-converting enzyme inhibitor; ARB, angiotensin receptor blocker.

### The TyG Index and Indicators of Cardiovascular Risk

**Figure 3:**
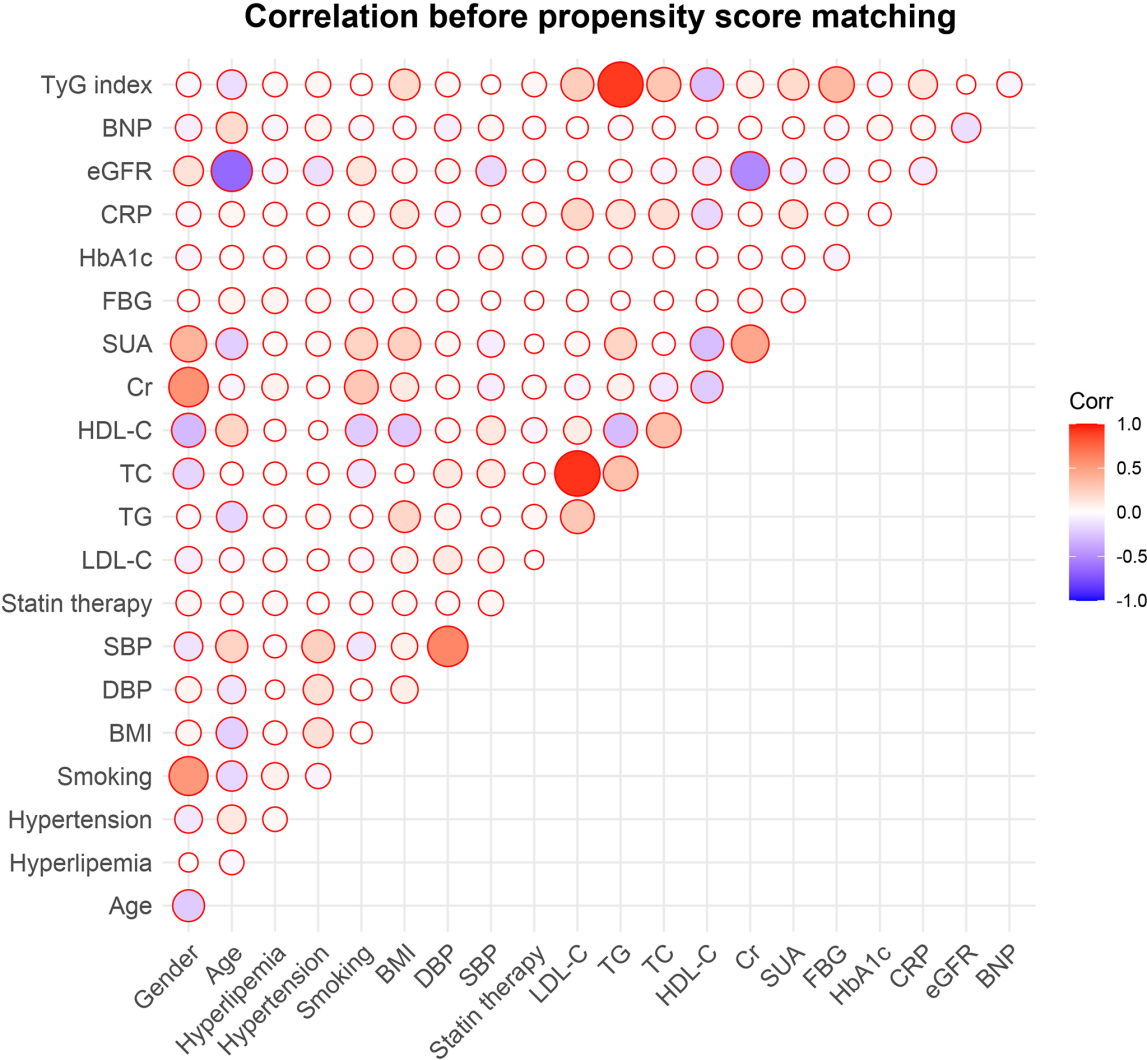
Correlations between the TyG index and traditional cardiovascular risk factors. TyG, triglyceride-glucose; BNP, brain natriuretic peptide; eGFR, estimated glomerular filtration rate; CRP, C-reactive protein; HbA1c, glycosylated hemoglobin A1c; FBG, fasting blood glucose; SUA, serum uric acid; Cr, creatinine; HDL-C, high density lipoprotein cholesterol; TC, total cholesterol; TG, triglyceride; LDL-C, low-density lipoprotein cholesterol; SBP, systolic blood pressure; DBP, diastolic blood pressure; BMI, body mass index.

The correlations between traditional cardiovascular risk indicators or commonly-used risk indicators of CVD and the TyG index were examined. The TyG index was positively linked to DBP, history of hyperlipemia, and smoking status (P<0.05) while correlating negatively with gender, HDL-C and BNP (P<0.05) (**Figure 3**).

### The TyG Index and MACCEs

**Figure 4:**
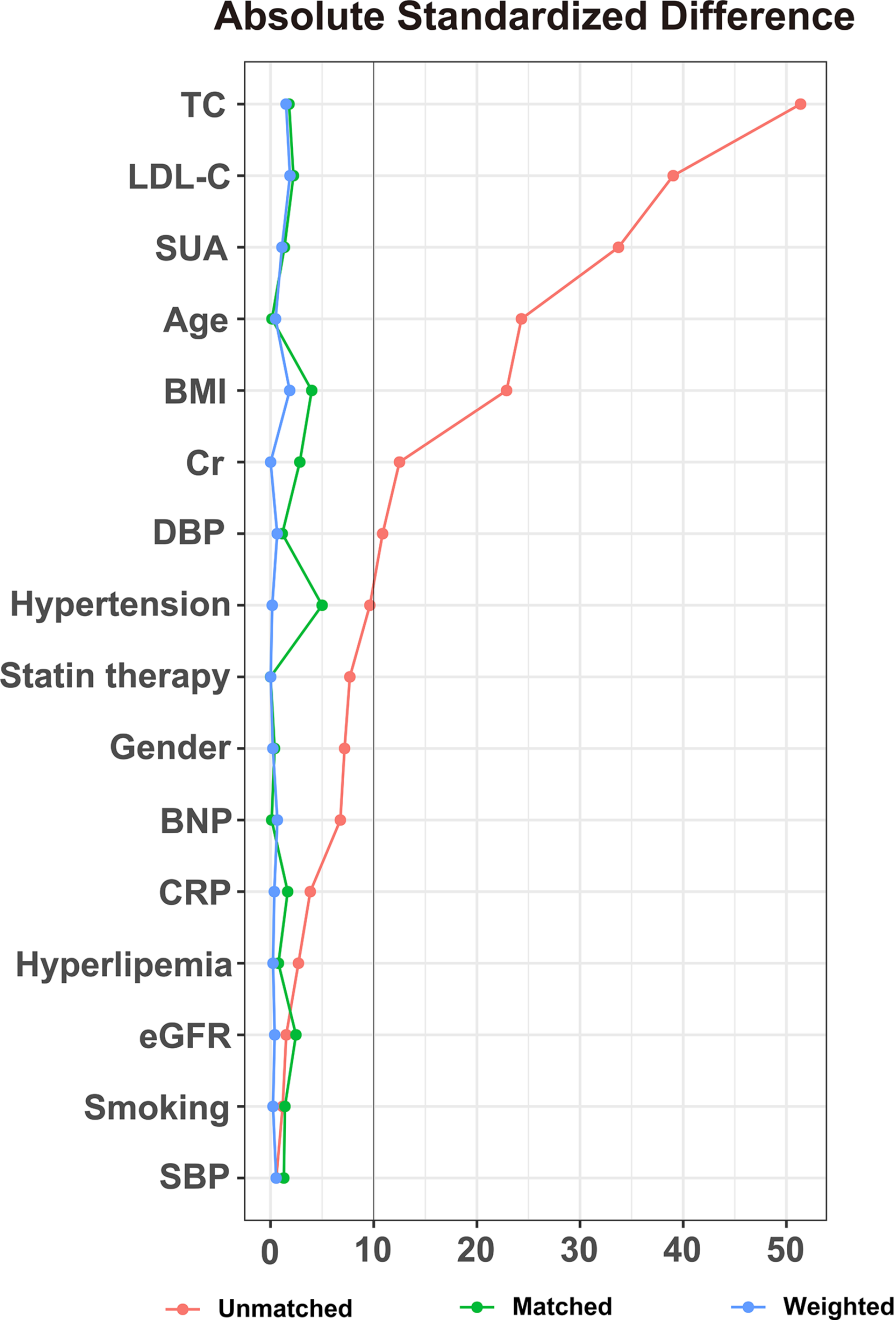
Absolute standardized differences in unweighted and propensity score-weighted data sensitivity analyses. TC, total cholesterol; LDL-C, low-density lipoprotein cholesterol; SUA, serum uric acid; BMI, body mass index; Cr, creatinine; DBP, diastolic blood pressure; BNP, brain natriuretic peptide; CRP, C-reactive protein; eGFR, estimated glomerular filtration rate; SBP, systolic blood pressure.

After the stratification of the MACCEs incidence, propensity score matching was performed (**Figure 4**). This showed significant differences between the low and high-TyG index groups in terms of gender, age, SBP, DBP, BMI, statin therapy, TC, SUA, LDL-C, Cr, BNP, CRP, eGFR, history of hyperlipemia, hypertension and smoking was not found.

#### Prediction of MACCEs Using the TyG Index

**Figure 5:**
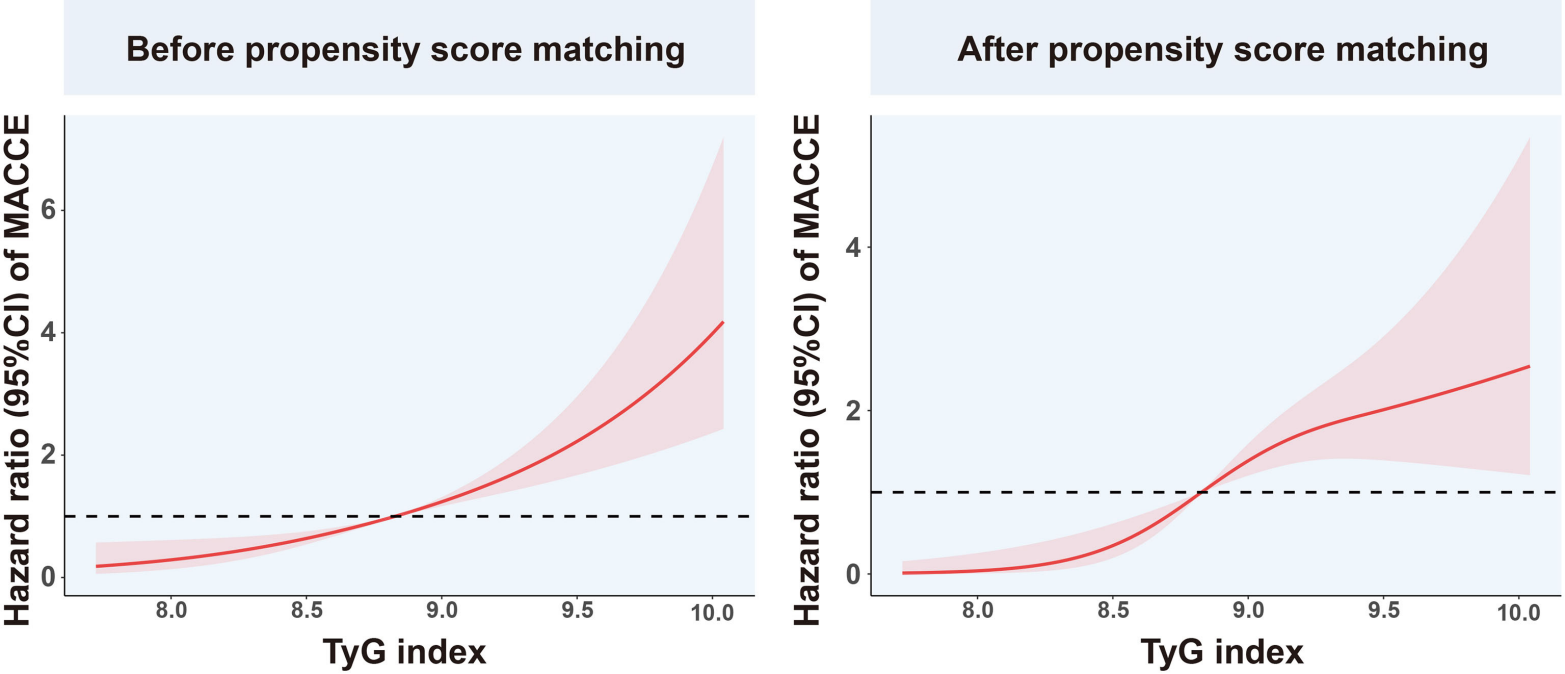
Restricted cubic spline analysis of the association between TyG index and MACCEs. TyG, triglyceride-glucose; MACCEs, major adverse cardiovascular and cerebrovascular events.

Among the overall population, in the univariate Cox proportional hazard analysis, the TyG index, Cr, and SUA were found to be independently related with MACCEs (P<0.05). In the multivariate analysis, the variables gender, age, BMI, LDL-C, HDL-C, SBP, DBP, TyG index, Cr, SUA, eGFR, BNP, CRP, history of smoking, hypertension, and hyperlipemia were adjusted. It was found that after adjustment, only the TyG index was independently related to MACCEs (**Table 3**). Using RCS, we evaluated the shape of the association using penalized splines to examine the relationship between MACCEs risk and the TyG index. In populations of pre/post propensity score matching, with increased TyG index, the RCS curves showed the same trend of monotonic increase in the risk of MACCEs (**Figure 5**).

**Table 3:** Independent predictors of MACCEs in overall patients.

Variables	Univariate analysis		Multivariate analysis	
	HR (95% CI)	P	HR (95% CI)	P
**Age**	0.989 (0.977-1.001)	0.073	1.009 (0.982-1.037)	0.498
**Male**	1.122 (0.829-1.519)	0.454	0.760 (0.412-1.401)	0.379
**BMI**	1.033 (0.995-1.072)	0.087	0.986 (0.944-1.030)	0.524
**SBP**	0.999 (0.991-1.007)	0.823	1.001 (0.990-1.013)	0.802
**DBP**	1.003 (0.991-1.015)	0.592	1.000 (0.983-1.017)	0.984
**Smoking**	1.115 (0.862-1.442)	0.409	1.092 (0.801-1.488)	0.579
**Hypertension**	1.014 (0.780-1.317)	0.920	0.984 (0.745-1.301)	0.903
**Hyperlipemia**	0.969 (0.734-1.278)	0.822	0.925 (0.698-1.225)	0.579
**LDL-C**	1.083 (0.933-1.256)	0.295	1.039 (0.532-2.031)	0.672
**HDL-C**	0.298 (0.171-0.519)	<0.001	0.873 (0.338-2.251)	0.778
**TyG index**	4.453 (3.507-5.653)	<0.001	4.942 (3.432-6.115)	<0.001
**Cr**	1.009 (1.001-1.018)	0.025	1.014 (0.986-1.042)	0.328
**SUA**	1.002 (1.001-1.004)	0.001	1.000 (0.999-1.002)	0.819
**eGFR**	0.999 (0.989-1.009)	0.829	1.015 (0.981-1.051)	0.378
**BNP**	1.000 (0.999-1.001)	0.906	1.000 (0.999-1.001)	0.955
**CRP**	1.010 (0.988-1.031)	0.378	1.009 (0.986-1.032)	0.458

HR, hazard ratio; CI, confidence level; BMI, body mass index; SBP, systolic blood pressure; DBP, diastolic blood pressure; LDL-C, low-density lipoprotein cholesterol; HDL-C, high density lipoprotein cholesterol; TyG, triglyceride-glucose; Cr, creatinine; SUA, serum uric acid; eGFR, estimated glomerular filtration rate; BNP, brain natriuretic peptide; CRP, C-reactive protein.

#### The TyG Index and Clinical Outcomes

**Figure 6:**
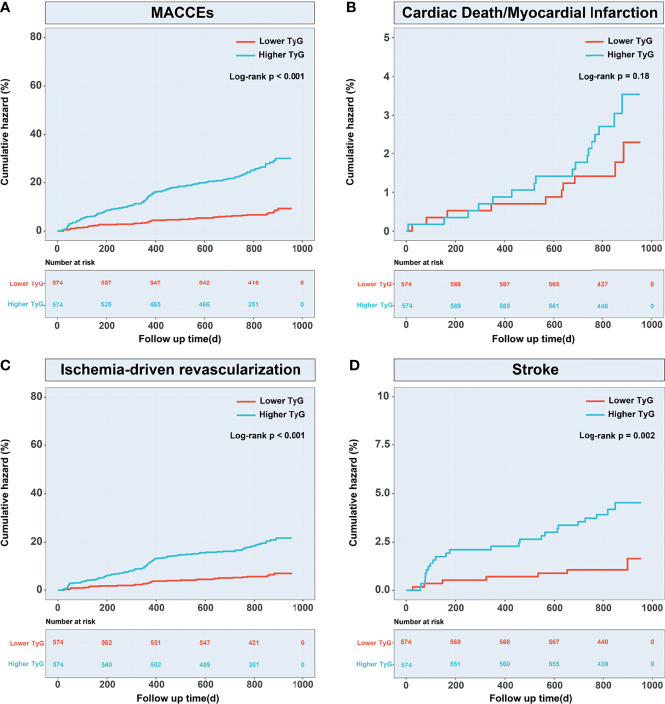
Kaplan–Meier curves for MACCEs and endpoint events according to the propensity score-matched population. **(A)** Kaplan–Meier curves for MACCEs; **(B)** Kaplan–Meier curves for cardiac death or non-fatal myocardial infarction; **(C)** Kaplan–Meier curves for ischemia-driven revascularization; **(D)** Kaplan-Meier curves for stroke. TyG, triglyceride-glucose; MACCEs, major adverse cardiovascular and cerebrovascular events.

The occurrence of MACCEs, ischemia-driven revascularization, and stroke differed significantly between the groups (P<0.01), shown by pre/post propensity score matching and Cox proportional hazard analyses (**Table 4**). Kaplan–Meier survival analysis confirmed a greater incidence of MACCEs (P < 0.001), ischemia-driven revascularization (P < 0.001), and stroke (P = 0.002) in the higher TyG index group (**Figure 6**).

**Table 4:** Estimated Kaplan–Meier events rates of 2.5 years follow-up.

	Overall population				Propensity score-matched population			
Adverse events	Lower TyG index (< 8.83, n = 1358)	Higher TyG index (≥8.83, n=672)	Adjusted HR (95% CI)	P	Lower TyG index (< 8.83, n = 574)	Higher TyG index (≥8.83, n=574)	HR (95% CI)	P
**MACCEs, n (%)**	77 (5.7)	156 (23.2)	4.942 (3.432-6.115)	<0.001	42 (7.3)	136 (23.7)	3.526 (2.618-4.749)	<0.001
**Cardiac death, n (%)**	8 (0.6)	3 (0.4)	0.652 (0142-2.997)	0.583	5 (0.9)	2 (0.3)	0.461 (0.099-2.138)	0.322
**MI, n (%)**	10 (0.7)	19 (2.8)	4.844 (2.060-6.388)	<0.001	7 (1.2)	15 (2.6)	2.278 (0.968-5.360)	0.059
**Cardiac death/MI, n (%)**	15 (1.1)	22 (3.3)	3.139 (1.470-6.703)	0.003	10 (1.7)	17 (3.0)	1.684 (0.773-3.673)	0.190
**Ischemia-driven revascularization, n (%)**	60 (4.4)	120 (17.9)	4.116 (3.612-6.246)	<0.001	34 (5.9)	104 (18.1)	3.455 (2.459-4.856)	<0.001
**Stroke, n (%)**	13 (1.0)	28 (4.2)	2.405 (1.647-3.307)	0.001	7 (1.2)	24 (4.2)	2.902 (1.431-5.885)	0.003

HR, hazard ratio; CI, confidence level; TyG, triglyceride-glucose; MACCEs, major adverse cardiovascular and cerebrovascular events; MI, myocardial infarction.

## Discussion

This appears to be the first investigation of the relationship between prognosis and the TyG index in prediabetic patients with ACS. Firstly, the study included 2030 patients with prediabetes and ACS who were followed up for two-and-a-half years during which the total incidence of MACCEs was 11.5%. After 1:1 propensity score matching, MACCEs incidence to be significantly greater in patients with high TyG indices, with ischemia-driven revascularization and stroke dominating. Secondly, multivariate Cox regression analysis of the overall population showed a significant link between the TyG index and MACCEs, and the RCS curve also indicated a consistent increase between the index and the HR both before and after matching.

### The TyG Index and Atherosclerosis

The TyG index has long been regarded as an IR indicator. IR in the liver is frequently estimated by the homeostasis model assessment of insulin resistance (HOMA-IR). Kim et al. concluded that TyG index was more effective HOMA-IR in predicting coronary artery calcification (CAC) (26), and Park et al. have also pointed out that the TyG index predicts CAC progression, especially in adults with non-severe CAC (27). IR has also been found to influence plaque development by promoting apoptosis in vascular smooth muscle cells, macrophages and endothelial cells (28). In addition, studies have shown that prediabetic patients have more severe coronary atherosclerosis and plaque vulnerability than non-diabetic patients (29). Considering the substantial impact of diabetes on CAC, these results may be related to the increased prevalence of diabetes and the increase in the TyG index (30), where the critical mechanisms may include the activation of endothelial dysfunction, vascular inflammation, and oxidative stress by hyperglycemic injury (31). An observational study reported that in asymptomatic diabetic patients, the CAC score can be used effectively to assess obstructive coronary artery disease (CAD) by coronary computed tomography angioplasty (CTA) (32). Subsequently, observing coronary plaques through CTA, Won et al. also found a correlation between the TyG index and severity of CAD, suggesting that IR accompanied by CAC affects the progression of coronary plaques (33).

### Prediction of MACCEs by the TyG Index in Prediabetic Patients

#### The TyG Index and Stroke

An association between the TyG index and increased probability of IR recurrence, neurological decline, and all-cause mortality has been found in patients with ischemic stroke (34). A higher TyG index can also predict a poor functional prognosis of acute ischemic stroke (35). In addition, the TyG index is able to forecast both the hospitalization and intensive care unit mortality after stroke, especially with ischemic stroke (36). Shi et al. considered that TyG could assist in the assessment of the probability of ischemic stroke (37). In addition, an increased TyG index independently predicts the risk of ischemic stroke in the general population, and IR may be positively correlated with future stroke risk (38). Although studies have found that the correlation between BMI and stroke prognosis is not affected by the TyG index (39), it is interesting that Du et al. reported an association between the TyG-BMI index derived from the TyG index and ischemic stroke (40).

A recent registration study observed a relationship between elevated higher TyG indices and increased MACCEs risk with STEMI, and concluded that the TyG index can effectively predict outcomes in STEMI cases after percutaneous coronary intervention (PCI) (41). Subsequently, Zhang et al., in examining the relationship between the TyG index and MACCEs, concluded that the former was valuable in assessing both prognosis and risk stratification in T2DM patients after suffering acute myocardial infarction (AMI) (42). In our research, we found a 2.0% incidence of stroke in all patients during the 2.5-year follow-up. After propensity score matching, survival analysis showed that the risk of stroke and the TyG index were significantly different between higher and lower TyG groups, which is consistent with previous studies. In addition, the higher TyG index group had a poorer prognosis (HR 2.902, 95%CI: 1.431-5.885, P<0.001), and it was shown that the TyG index may be closely linked to the occurrence of stroke in prediabetic patients with ACS.

#### The TyG Index and Cardiovascular Events

Evidence shows that the TyG index is predictive of cardiovascular events. Perusal of the recent literature shows that there is similar evidence in different populations of CAD. Firstly, in SAP patients, the TyG index correlated positively with cardiovascular events, indicating that TyG is a valuable indicator in predicting the clinical outcome of CAD patients (43). Concurrent studies confirmed that in SAP patients with T2DM, compared with the hemoglobin glycation index, the TyG index is superior in terms of prognostic value (44).

In the ACS population, it has been found that high TyG indices may be related to a greater risk of major adverse cardiovascular events (MACE) in AMI patients (45). A recent study has shown that in NSTEMI patients, MACE occurred more frequently in patients with high TyG indices (46). In addition, the TyG index was also found to predict future MACE in patients with ACS (47).

Previous studies have shown that compared with HbA1c and TG, the TyG index is a valuable forecaster of future cardiovascular events, and may provide additional prognostic benefits for T2DM (48). For ACS patients treated with PCI, compared with FPG or HbA1c, the TyG index may be superior in predicting cardiovascular events (49). In non-diabetic patients, a higher TyG index predicts future ischemic heart disease, indicating that it may be a valuable indicator for assessing the risk of cardiovascular events in non-diabetic adults (50). Similarly, in non-diabetic ACS patients, higher TyG indices are associated with a greater incidence of revascularization or AMI and larger infarct size (51), suggesting that high TyG indices can effectively predict revascularization. However, few studies have addressed the prognosis of prediabetic patients with ACS. The existing studies only report a causal relationship between prediabetes combined with all-cause mortality and CVD (29), with the TyG index recognized as potentially useful in the early detection of patients at risk of developing CVDs and adverse outcomes (52). In addition, prediabetes as a metabolic disorder might affect the MACCEs *via* over-inflammation and oxidative stress through inflammatory/oxidative stress pathways at the levels of cardiac (fat) tissue and the atherosclerotic plaque (12, 13, 53). The same pathways could be more evidenced in over-weight subjects and influence the epigenetic, the MACEs, and be influenced (positively) by metformin therapy (54). Moreover, prediabetic patients have alteration of inflammatory markers and of the value of endothelial function that consequently could cause higher rate of MACEs also in absence of significant coronary stenosis (55). In summary, our study indicates a close link between the TyG index and MACCEs in patients with prediabetes and ACS, with higher TyG indices related to poor prognosis. As the levels of the TyG index are rising, this may be a prognostic indicator for prediabetic patients with ACS.

## Conclusions

The TyG index is an important simple composite index of IR in prediabetic patients, and high TyG indices may be significant prognostic indicators in prediabetic patients with ACS.

## Limitations

(1) This is a single center study with a small sample size, and more multi-center studies need to be performed to confirm the present results. In addition, the retrospective nature of our study potentially biases the result of analysis. However, the prevalence of clinical risk factors in our study population was similar to some contemporary trials and real-world registries (56, 57). This might be a relevant finding and potentially supports the generalizability of our results. (2) TyG indices were only calculated at admission for only once and might change in the 2.5-year follow-up. However, we were not able to measure changes in TyG indices over time. (3) Due to lack of fasting insulin data, the calculation of HOMA-IR was not performed in the present study. (4) Since our study mainly focused on the presence of MACCEs in patients during the follow-up, we did not carry out subsequent hematological examination on these patients, and it was not possible to determine whether the patients progressed from prediabetes to diabetes or non-diabetes. (5) Outcome events in this study were not adjudicated by a clinical events committee. This represents a possible limitation of results that should be acknowledged (58).

## Data Availability Statement

The data supporting the conclusions of this study will be available from the corresponding authors on reasonable requests.

## Ethics Statement

The studies involving human participants were reviewed and approved by the Institutional Ethics Committee of Beijing Anzhen Hospital. The data retrospectively obtained from electronic medical records.

## Author Contributions

QYG, XXF, BZ, and GYZ made contributions to the acquisition of data, analysis and drafting of the manuscript. QYG, XXF, YL, and JQY made contributions to the acquisition and interpretation of data. QYG, YJZ and DMS made substantial contributions to conception and design. YYL and YJZ made substantial contributions to critical revision of the manuscript. All authors read and approved the final manuscript.

## Funding

This study was supported by the grant from Natural Science Foundation of Beijing, China (Grant No. 7214223) to QG. YZ was supported by National Key Research and Development Program of China (2017YFC0908800), Beijing Municipal Health Commission (Grant No. PXM2020_026272_000002 and Grant No. PXM2020_026272_000014) and Natural Science Foundation of Beijing, China (Grant No. 7212027).

## Conflict of Interest

The authors declare that the research was conducted in the absence of any commercial or financial relationships that could be construed as a potential conflict of interest.

## Publisher’s Note

All claims expressed in this article are solely those of the authors and do not necessarily represent those of their affiliated organizations, or those of the publisher, the editors and the reviewers. Any product that may be evaluated in this article, or claim that may be made by its manufacturer, is not guaranteed or endorsed by the publisher.

## Abbreviations

T2DM, type 2 diabetes; IR, insulin resistance; CVDs, cardiovascular diseases; TyG, triglyceride-glucose; ACS, acute coronary syndrome; MACCEs, major adverse cardiovascular and cerebrovascular events; HbA1c, glycosylated hemoglobin A1c; UAP, unstable angina pectoris; NSTEMI, non-ST-segment elevation myocardial infarction; STEMI, ST-segment elevation myocardial infarction; CABG, coronary artery bypass grafting; ROC, receiver operating characteristic; BMI, body mass index; SBP, systolic blood pressure; DBP, diastolic blood pressure; TG, triglyceride; FBG, fasting blood glucose; TC, total cholesterol; LDL-C, low-density lipoprotein cholesterol; HDL-C, high density lipoprotein cholesterol; Cr, creatinine; SUA, serum uric acid; CRP, C-reactive protein; hs-TNI, high sensitivity troponin I; MI, myocardial infarction; IQR, interquartile range; HRs, hazard ratios; RCS, restricted cubic spline; CI, confidence level; ACEI, angiotensin-converting enzyme inhibitor; AUC, area under curve; BNP, brain natriuretic peptide; eGFR, estimated glomerular filtration rate; HOMA-IR, Homeostasis model assessment of insulin resistance; CAC, coronary artery calcification; CAD, coronary artery disease; CTA, computed tomography angioplasty; ICU, intensive care unit; PCI, percutaneous coronary intervention; AMI, acute myocardial infarction.
