# Supplementary material for: Influence of the Triglyceride-Glucose Index on Adverse Cardiovascular and Cerebrovascular Events in Prediabetic Patients With Acute Coronary Syndrome
Source: Front Endocrinol (Lausanne). 2022 Feb 22;13:843072. doi: 10.3389/fendo.2022.843072 (PMC8920560; doi:10.3389/fendo.2022.843072)
Supplement: Supplementary file 1 [file DataSheet_1.docx]

Supplementary material

1. Methods of propensity score-matched analysis

According to the ROC, the optimal cutoff point of the TyG index was identified, and patients were divided into 2 groups. Propensity score matching analysis was performed in the two groups with a proportion of 1:1.

2. Detailed results of propensity score-matched analysis

| variables | Lower TyG index  n=573 | Higher TyG index  n=573 | P | test SMD |
| --- | --- | --- | --- | --- |
| Age, years | 58.07±10.49 | 58.11±9.98 | 0.940 | 0.004 |
| Gender, (male%) | 414 (72.1) | 414 (72.1) | 1.000 | <0.001 |
| BMI, kg/m2 | 25.93±3.69 | 25.83±2.97 | 0.604 | 0.031 |
| SBP, mmHg | 127.85±16.70 | 128.25±16.37 | 0.679 | 0.024 |
| DBP, mmHg | 77.30±11.65 | 77.38±10.66 | 0.903 | 0.007 |
| Medical history, n (%) |  |  |  |  |
| Smoking | 287 (50.0) | 281 (49.0) | 0.768 | 0.021 |
| Hypertension | 351 (61.1) | 356 (62.0) | 0.808 | 0.018 |
| Hyperlipemia | 399 (69.5) | 401 (69.9) | 0.949 | 0.008 |
| Laboratory results |  |  |  |  |
| LDL-C, mmol/L | 2.53±0.90 | 2.50±0.77 | 0.445 | 0.045 |
| TC, mmol/L | 4.21±0.99 | 4.18±0.88 | 0.645 | 0.027 |
| Cr, μmol/L | 70.80±14.43 | 71.28±15.67 | 0.590 | 0.032 |
| SUA, μmol/L | 362.49±88.76 | 362.92±88.67 | 0.935 | 0.005 |
| eGFR, mL/(min* 1.73 m2) | 97.01±12.93 | 96.50±13.79 | 0.516 | 0.038 |
| BNP, pg/mL | 45.56±88.00 | 44.69±105.75 | 0.879 | 0.009 |
| CRP, mg/L | 3.22±5.56 | 3.12±5.03 | 0.758 | 0.018 |
